# Diastolic Heart Murmur, Nocturnal Back Pain, and Lumbar Rigidity in a 7-Year Girl: An Unusual Manifestation of Lyme Disease in Childhood

**DOI:** 10.1155/2012/976961

**Published:** 2012-09-29

**Authors:** Genn Kameda, Silvia Vieker, Johannes Hartmann, Tim Niehues, Alfred Längler

**Affiliations:** ^1^Department for Child and Adolescent Health, Gemeinschaftskrankenhaus, Gerhard-Kienle Weg 4, 58313 Herdecke, Germany; ^2^Consultant for Pediatric Cardiology, Gemeinschaftskrankenhaus, Gerhard-Kienle Weg 4, 58313 Herdecke, Germany; ^3^Center for Child and Adolescent Health, Helios Klinikum, Lutherplatz 40, 47805 Krefeld, Germany; ^4^University of Witten/Herdecke, Faculty of Health, Center for integrative Medicine, Alfred-Herrhausenstr. 50, 58448 Witten, Germany

## Abstract

A 7-year-old girl presented with nocturnal pain in her back and legs. The physical examination revealed a loud opening sound of the mitral valve and lumbar rigidity. With the exception of significantly increased anti-nuclear antibody (ANA) levels, the immunological findings did not show any other abnormal parameters, also spinal magnetic resonance imaging (MRI) and ultrasound examination of the abdomen and pelvis yield no pathological findings. The lumbar puncture showed a lymphocytic pleocytosis as well as an intrathecal synthesis of Borrelia-specific antibodies. Echocardiography showed a thickened mitral valve with mild regurgitation. No other signs of florid endocarditis or myocarditis could be detected. Due to these findings, the diagnosis Lyme neuroborreliosis was made and an intravenous antibiotic therapy was given. The clinical symptoms subsided. Six months later, she had an almost normal mitral valve with only trivial mitral insufficiency. The association between the lumbar rigidity and the thickened mitral valve remains unclear. The case of our patient with nocturnal back and leg pain may be considered a rare case of Lyme neuroborreliosis with meningoradiculitis in children, and to our knowledge these symptoms together with cardiac involvement, such as a significantly thickened mitral valve, have not yet been described in the literature.

## 1. Introduction

In children, Lyme disease may occur as early localized, early disseminated, or as late chronic manifestation after the tick bite, respectively [1]. Neurological symptoms occur quite often and may involve cranial nerves in up to 10% mostly as facial palsy [2]. Cardiomyopathy as initial symptom in children has been reported in single cases [3, 4] but observed in adults in 4–8% [5]. We report on a child with nocturnal pain of the back and the legs and a transient cardiac murmur as the only symptoms of an infection with *Borrelia burgdorferi*. 

## 2. Case Report

A 7-year-old girl was presented with nocturnal back and leg pain for the past 6 weeks. A tick bite in the popliteal fossa was observed 8 weeks before onset of the symptoms, but no erythema migrans. The physical examination revealed a diastolic heart murmur (3/6), suspected to be an opening sound of the mitral valve. The examination of the locomotor organs showed a lumbar rigidity with a pathological finger floor-distance of 50 cm together with a positive Lasègue sign without any other neurological symptoms.

**Figure 1:**
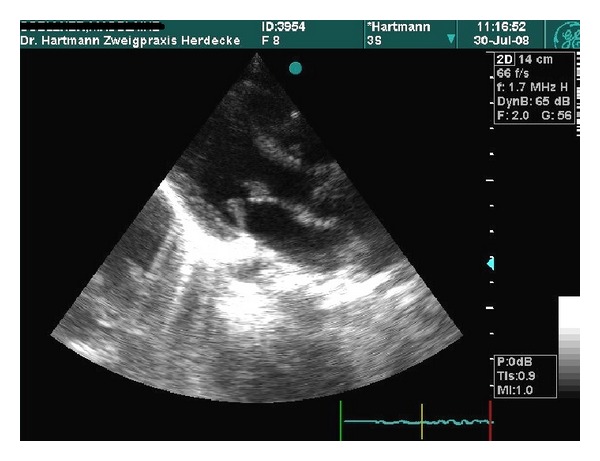
Picture showing a long axis view of the heart during diastolic opening of the mitral valve: notice the terminal clubbed thickness of both mitral valves.

The laboratory examinations (blood cell count, creatinine, urea, protein, aspartate and alanine aminotransferase, bilirubine, prothrombin, thromboplastin, and c-reactive protein) were normal. There was no proof of bacteria in blood cultures or throat swabs. Serological examinations for Cytomegalovirus, Epstein-Barr virus, and Coxsackievirus and for antistreptolysin, Mycoplasma pneumonia, and *Chlamydia pneumoniae* were negative. The serum was positive for *Borrelia burgdorferi* immunoglobulin M (IgM) und IgG antibodies (ELISA and immunoblot). In the cerebrospinal fluid (84/ul leucocytes, protein 113 mg/dL), an intrathecal borrelia IgM (33.7, normal: <1.5) and IgG (15.4, normal: <1.5) antibody synthesis was found. Immunoglobulins (IgG, IgA, IgM, IgE) and complement factors (C3, C4, CH50) were in normal range. The patient is HLA-B27 negative. Anti-nuclear antibodies (ANA) were elevated (1 : 640). Extractable nuclear antibodies and antibodies for double-stranded DNA were negative. The abdominal ultrasound and the magnetic resonance imaging of the pelvis revealed no pathological signs. The echocardiography (ECG) showed thickening of both mitral valve cups with a mild mitral insufficiency (I°) (Figure 1) and the electrocardiogram regular sinus rhythm with a physiologically incomplete right bundle branch block.

Due to these findings, the diagnosis Lyme neuroborreliosis could be verified and the thickening of the mitral valve cups was assumed to be a cardiac manifestation of Lyme disease. An intravenous therapy with Cefotaxime (200 mg/kd/d) was given over a period of 14 days.

**Figure 2:**
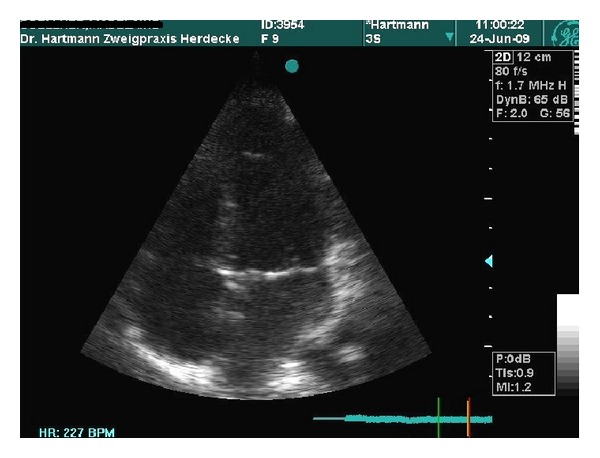
Four chamber view. 1 year later: mitral valve without thickness.

After 4 days of treatment, an improvement of the nocturnal symptoms was achieved and lumbar rigidity had significantly improved as well. After four weeks, there was still some thickening on the mitral valve. After 6 months, the patient was free of symptoms. The echocardiography showed terminal thickening of both cups of the mitral valve and only discrete mitral insufficiency. Twelve months later, the echocardiography showed an almost normal mitral valve with only trivial mitral insufficiency (Figure 2). The patient was free of symptoms.

## 3. Discussion

Lyme disease may have various forms of manifestation [1]. In children, meningoradiculitis and cardiomyopathy are known but rare [3], whereas in adult patients meningoradiculitis and myopericarditis are seldom seen as signs of early dissemination of Lyme disease [6].

In the literature, only very few case reports of carditis in children can be found [4] Unfortunately, exact data of patients with acquired heart diseases are not existent. A registrar in analogy to the German PAN Study (registration of congenital heart defects) may be useful to clarify correlations in rare cases with similar observations [7]. 

Recently, 207 children with early-disseminated Lyme disease were evaluated. Thirty-three (16%) had carditis. Of these patients, 14 (42%) had advanced heart block, including 9 (27%) with complete heart block. Out of the patients with carditis, 25 patients received further evaluation with echocardiography, but none of these had anatomical abnormalities, although 4 (12%) patients had depressed ventricular systolic function [3]. In three cases, complete heart block and shock led to endomyocardial biopsies, which revealed florid myocarditis. The incidence of Lyme carditis in other paediatric studies differs and tends to be lower [4, 5]. The nine published case reports or series with in total 14 children with Lyme carditis showed pathologic ECG findings such as atrioventricular block, long-corrected QT time or complete heart block, although normal ventricular function and no abnormalities of the cardiac valves were seen [3]. Endocarditis in adults seems to be also very rare as well and the occurrence of endocarditis and positive borrelia antibodies can lead to diagnostic confusions and misdiagnoses [8]. Therefore isolated endocarditis seems not to be a probable symptom of Lyme carditis in early disseminated stage.

We hypothesize that signs of an early manifestation of Lyme disease like meningoradiculitis and signs of a late manifestation like the changes of the mitral valve may occur simultaneously. Infections can trigger inflammatory reactions in patients with a genetic predisposition for autoinflammatory/autoimmune disease [9].

The classical example is Rheumatic fever where infection with streptococcus pyogenes is leading to arthritis and/or carditis with molecular mimicry being the suggested pathomechanism [10]. 

## 4. Conclusion

In summary, we describe the simultaneous presence of a Lyme neuroborreliosis and changes of the mitral valve. The authors conclude that it appears useful to do a cardiac examination including ECG and echocardiography in patients with Lyme neuroborreliosis and systolic heart murmur, and in case of nocturnal back pain with lumbar rigidity, neuroborreliosis should be ruled out.

## References

[1] Christen HJ (1996). Lyme neuroborreliosis in children. *Annals of Medicine*.

[2] Brunner J, Moschovakis G, Prelog M, Walder G, Wuerzner R, Zimmerhackl LB (2010). Lyme neuroborreliosis: aetiology and diagnosis of facial palsy in children from Tyrol. *Klinische Padiatrie*.

[3] Costello JM, Alexander ME, Greco KM, Perez-Atayde AR, Laussen PC (2009). Lyme carditis in children: presentation, predictive factors, and clinical course. *Pediatrics*.

[4] Woolf PK, Lorsung EM, Edwards KS (1991). Electrocardiographic findings in children with Lyme disease. *Pediatric Emergency Care*.

[5] Skogman BH, Croner S, Nordwall M, Eknefelt M, Ernerudh J, Forsberg P (2008). Lyme neuroborreliosis in children: a prospective study of clinical features, prognosis, and outcome. *Pediatric Infectious Disease Journal*.

[6] Steere AC, Batsford WP, Weinberg M (1980). Lyme carditis: cardiac abnormalities of Lyme disease. *Annals of Internal Medicine*.

[7] Lindinger A, Schwedler G, Hense HW (2010). Prevalence of congenital heart defects in newborns in Germany: results of the first registration year of the PAN study (July 2006 to June 2007). *Klinische Padiatrie*.

[8] Kaell AT, Volkman DJ, Gorevic PD, Dattwyler RJ (1990). Positive Lyme serology in subacute bacterial endocarditis. A study of four patients. *Journal of the American Medical Association*.

[9] Kamradt T, Göggel R, Erb KJ (2005). Induction, exacerbation and inhibition of allergic and autoimmune diseases by infection. *Trends in Immunology*.

[10] Froude J, Gibofsky A, Buskirk DR, Khanna A, Zabriskie JB (1989). Cross-reactivity between streptococcus and human tissue: a model of molecular mimicry and autoimmunity. *Current Topics in Microbiology and Immunology*.

